# *“My entire life has moulded the person that I am”*: Narrations of non‐suicidal self‐injury and complex trauma in individuals with complex posttraumatic stress experiences

**DOI:** 10.1111/papt.12583

**Published:** 2025-03-01

**Authors:** Reem Alharbi, Susanne Langer, Cheryl Hunter, Nusrat Husain, Filippo Varese, Peter James Taylor

**Affiliations:** ^1^ Department of Psychology King Abdulaziz University Jeddah Saudi Arabia; ^2^ Division of Psychology and Mental Health University of Manchester Manchester UK; ^3^ Complex Trauma and Resilience Research Unit Greater Manchester Mental Health NHS Foundation Trust Manchester UK; ^4^ Department of Psychology Manchester Metropolitan University Manchester UK; ^5^ University Hospitals Plymouth NHS Trust Plymouth UK

**Keywords:** complex PTSD, NSSI, self‐harm, self‐injury, thematic narrative analysis

## Abstract

**Background:**

Previous research suggests that complex trauma and Complex Posttraumatic Stress Disorder (C‐PTSD) experiences can contribute to the risk of developing and possibly maintaining Non‐Suicidal Self‐Injury (NSSI). Individuals' accounts of how complex trauma and subsequent development of C‐PTSD experiences can contribute to the difficulties of NSSI remain underexplored. This qualitative study aimed to explore in‐depth: (1) how individuals with C‐PTSD experiences narrate life conditions and events that influenced their difficulties with NSSI over time and (2) what factors the individuals perceived to have helped the process of controlling their experience of NSSI.

**Methodology:**

This novel qualitative narrative study used an adapted version of the Free Association Narrative Interviewing Method (FANIM) to facilitate the exploration of the lived experiences of eight individuals aged 20–56 years. The initial data analysis involved an interpretation of individuals' stories followed by a thematic narrative analysis of 14 interviews to explore the shared and unique experiences narrated by participants.

**Results:**

Four primary themes were established: (1) Voiceless, invisible, and out of control within the dysfunctional system during childhood, (2) “shaky foundation” leading to future traumas, (3) the link between complex trauma, mental health difficulties, and NSSI, and (4) Regaining autonomy and a sense of control in managing NSSI. The findings highlight the importance of adopting a flexible and person‐centred treatment that addresses the specific needs of these individuals. The treatment plan should empower individuals to improve their control and autonomy and support them to live a meaningful life.

## INTRODUCTION

Non‐suicidal self‐injury (NSSI) is defined as deliberate harm inflicted on one's own body without suicidal intent, and it involves cutting, burning, hitting, and scratching (Klonsky, 2007). In England, a prevalence rate of NSSI in the adult population of 6.4% has been estimated (McManus et al., 2016). NSSI is a serious public health concern as it is associated with an increased risk of future suicidal thoughts and behaviours (Glenn & Klonsky, 2013; Kiekens et al., 2018; Ribeiro et al., 2016) and is often an indicator of underlying mental health issues (Alharbi et al., 2020; Knorr et al., 2016; Nada‐Raja & Skegg, 2011). Approximately, 69% of people with NSSI reported at least one psychiatric diagnosis (Liu, 2023).

The association between childhood interpersonal trauma and NSSI is well‐supported. Up to 79% of children and adolescents with NSSI have a history of childhood trauma (Yates, 2009). Complex trauma, which is characterised by repeated and prolonged exposure to traumatic experiences (primarily interpersonal) that occur at a developmentally critical time (e.g., childhood maltreatment; Herman, 1992), is associated with a greater risk of NSSI (Christoffersen et al., 2015; Di Pierro et al., 2012). Studies in this regard have also found that exposure to complex trauma in the form of multiple interpersonal traumas (sexual and physical abuse) increases the risk of NSSI frequency (Di Pierro et al., 2012) compared to other types of traumas (e.g., death of a close relative; Christoffersen et al., 2015).

Childhood interpersonal trauma (complex trauma) has been linked to the development of Complex Posttraumatic Stress Disorder (C‐PTSD) (World Health Organization, 2019). C‐PTSD shares the core criteria of Posttraumatic Stress Disorder (PTSD; World Health Organization, 2019); however, it is distinguished from PTSD by the presence of three additional symptom clusters known as disturbance in self‐organisation (DSO): (1) negative views on self, (2) emotion dysregulation, and (3) interpersonal difficulties (Hyland et al., 2017).

There is a paucity of research that examines the direct link between C‐PTSD symptomology and NSSI. However, previous research suggests that C‐PTSD‐related difficulties such as PTSD and DSO symptoms are important factors associated with the development and maintenance of NSSI. PTSD symptoms were found to be potential mechanisms underlying the relationship between childhood interpersonal trauma and NSSI (Andersson et al., 2022; Howard et al., 2017; Weierich & Nock, 2008). A common function of NSSI is to avoid or escape psychological distress (Taylor et al., 2018, 2023), and hence NSSI may develop as a way to cope with distressing PTSD‐related experiences such as intrusions and hyperarousal (Alharbi et al., 2020).

Using risky ways of coping like NSSI may contribute to maintaining PTSD symptoms when self‐injury is used as a way to avoid talking or thinking about trauma reminders (Murray & El‐Leithy, 2022). Similarly, aspects of the DSO such as emotion dysregulation (Andersson et al., 2022; Liu et al., 2022; Titelius et al., 2018; Yang et al., 2022), negative self‐concept and related feelings of shame, self‐blame, and self‐criticism (Gandy, 2014; Glassman et al., 2007; Swannell et al., 2012; Vieira et al., 2018), and interpersonal difficulties (He & Xiang, 2022) also play a mediating role between childhood interpersonal trauma and NSSI. These results are consistent with several theories of NSSI (Hooley & Franklin, 2018; Nock, 2009), including the cognitive‐emotional theory of NSSI (Hasking et al., 2017), which highlights how negative self‐perception and the function of regulating emotional states may drive NSSI. Likewise, the benefits and barriers model of NSSI argues that self‐esteem represents an important barrier to self‐injury (Hooley & Franklin, 2018), and this may become eroded in people with C‐PTSD.

The current evidence relies on quantitative, mostly cross‐sectional, designs. Qualitative approaches provide a means of further elucidating these associations, enabling an in‐depth exploration of how complex trauma increases the risk of NSSI, especially in people experiencing C‐PTSD difficulties. The open‐ended nature of qualitative inquiry may be valuable in allowing unexpected findings and observations to emerge. Past qualitative research has highlighted how self‐injury has been a response to trauma for some, providing a means to regulate, escape, or communicate the emotional (or dissociated) states that have followed traumatic experiences (Harris, 2000; Stänicke et al., 2018). This mirrors wider research around the functional nature of NSSI (Taylor et al., 2023). Qualitative research has also identified how being caught in disempowered or conflictual relational states contributes to self‐harm, highlighting how the wider interpersonal context of trauma may be important (Peel‐Wainwright et al., 2021). Whilst qualitative research has taken various approaches, narrative methods (Hollway & Jefferson, 2000) may be particularly useful in untangling the complex histories of people who have experienced chronic trauma and understanding how this links to their self‐injury.

The relationship between complex trauma, C‐PTSD, and NSSI is likely dynamic and evolves over time as individuals develop different ways of coping with the aftermath of trauma (Murray & El‐Leithy, 2022). A recent study on the process of change in NSSI suggested a complex and non‐linear process that evolves over time and is linked to various external and internal variables (Kruzan & Whitlock, 2019). To the authors' knowledge, no previous qualitative studies have studied individuals' accounts of how complex trauma and C‐PTSD experiences are linked to NSSI. The study aims to understand in‐depth how individuals with C‐PTSD experiences narrate life conditions and events that influenced their difficulties with NSSI and what factors the individuals perceived to have helped the process of controlling their experience of NSSI.

## METHOD

### Design

We used techniques adapted from the Free Association Narrative Interview Method (FANIM; Hollway & Jefferson, 2000). The method was designed to capture the complex and emotionally charged experiences over a period of time using a single open‐ended question asking for a life story of particular experiences in the initial exploration interview (Hollway & Jefferson, 2000). Without interruption by the interviewer, individuals were given the opportunity to narrate their stories in the same sequence they happened in the past (Wengraf, 2008). This method allows for in‐depth exploration of complex and temporal experiences, which may not be possible using traditional interview methods (Wengraf, 2008). The initial exploration is followed up with a semi‐structured interview designed to explore in depth the participant story mentioned in the first interview (Hollway & Jefferson, 2000). A safety protocol was developed for this study to ensure the safety and well‐being of participants. This encompassed a wide variety of responses, both within the interview (e.g., pausing or ending) and beyond (e.g., signposting, calling emergency services) that varied based on context and level of risk (a copy is available on reasonable request). Before taking part, safety considerations were discussed and agreed upon with all participants. This study was approved by an NHS Research Ethics Committee (Northwest‐ Greater Manchester West REC, Ref: 21/NW/0047).

### Participants

Recruitment took place between May 2021 and September 2022. Participants were purposively recruited through various NHS mental health services and third‐sector mental health organisations in the Northwest of England. Recruitment from a university in the Northwest of England also took place. Participants were either referred by their clinicians or self‐referred via the study adverts placed in the waiting rooms of participating services or posted on the study's Twitter account and university email bulletins. The eligibility criteria were: (1) engaging in NSSI on five or more days at any point in the person's life; (2) having a history of trauma or a current diagnosis of PTSD as confirmed by their clinical team; (3) being 16 years old or older; (4) having adequate English language comprehension skills to read study materials and understand the researcher; (5) receiving support from mental health services at the time of recruitment; and (6) meeting C‐PTSD criteria at the time of eligibility checking. Eligibility was assessed via self‐report measures during a screening phone call with participants. The Life Events Checklist (LEC‐5; Weathers et al., 2013) and the International Trauma Questionnaire (ITQ; Cloitre et al., 2018) were used to determine the presence of C‐PTSD. The LEC was used in tandem with the ITQ to first identify if traumatic experiences were present and then as an anchor for the ITQ questions regarding C‐PTSD experiences. Individuals were initially excluded from the study if there was a judged high or imminent risk of harm to themselves, operationalised as having current suicidal ideation with an active plan or had recently engaged in medically serious NSSI (e.g., ligaturing, overdose) that required hospitalisation.

### Measures

In addition to the demographic questionnaire, which we used to collect general information about participants (i.e., age, ethnicity, gender, employment status and clinical mental health history), we utilised the following measures:

1. *Self‐Injurious Thoughts and Behaviours Interview (STIBI)‐short‐version*: The STIBI is a structured interview that assesses features of a broad spectrum of self‐injurious thoughts and behaviours. Only NSSI and suicidal attempt subsections were used in this study. The STIBI is a widely used measure in NSSI research and has good validity and reliability psychometric properties (Nock et al., 2007).
2. *Life Events Checklist (LEC‐5)*: This questionnaire was used to assess the occurrence of sixteen potentially traumatic events across participants' lives. The measure exhibited good reliability and validity (Weathers et al., 2013). Consistent with previous research (Resick et al., 2015), we only reported events that participants rated as having personally experienced or witnessed.
3. *International Trauma Questionnaire (ITQ)*: This is a self‐report measure which assesses the core symptoms of PTSD and C‐PTSD and was developed in accordance with the diagnostic criteria of C‐PTSD, which is presented in the 11th edition of the International Classification of Diseases (ICD‐11; World Health Organisation, 2019) (see Cloitre et al., 2018 for information about the diagnostic scoring of the tool). The measure is valid and reliable in all C‐PTSD symptom clusters (*α* range = .80 to .94; Shevlin et al., 2018).

### Interviews

#### First interview

The first interview involved asking the participant a single open‐ended narrative question about their story of NSSI. The format of this question was adapted from Wengraf (2008). Participants were asked the following question: “In this interview, I am interested in hearing about your story of self‐injury. In particular, I would like you to tell me about how your life experiences up until now have affected your self‐injury and the way this has developed. Start wherever you like, and please take the time you need. I won't interrupt. I will just make some notes for themes I will ask you about when we meet again after a week from now. Is that okay?”. The interviewer listened actively without interruption, using minimal verbal and nonverbal cues. The interview ends when the participants end their story and have nothing else to say. The first interview was used as a guide for the follow‐up (semi‐structured) interview, which was conducted approximately a week from the first. The first interview lasted 10–40 min (mean = 25 min). All participants completed the first interview.

#### Follow‐up (second) interview

The purpose of this interview was to allow the participant to ask follow‐up questions to gain more information about unclear aspects of their story and allow participants to further elaborate on their accounts. A topic guide was developed containing specific questions and relevant prompts, which were unique and tailored to each participant. However, the questions covered areas relevant to the research question (e.g., changes in NSSI over time, factors and life experiences linked to this change, and helpful ways to manage NSSI). The questions were written using participants' own words and followed the same order as topics were raised in the first interview, to preserve the narrative flow. All questions were designed by the first author and further reviewed by a member of the research team prior to the interview. The duration of this interview was between 40 and 60 min (mean = 50 min). Two participants did not proceed with the second interview. One found the participation difficult and decided to stop. The second participant was considered at high risk of self‐harm following the first interview. Following our safety protocol, we consulted with the participant's clinical team, and it was advised it would not be in the participant's best interest to proceed with the second interview.

All interviews were conducted by the first author (lead researcher). Seven participants chose to be interviewed via video call, and only one preferred to meet face‐to‐face for both study interviews. All interviews were audio‐recorded and transcribed verbatim. All transcripts were revised to check their quality by re‐listening the audio recordings.

### Analytical approach

Data analysis was conducted in two stages. The first stage involved reading through the transcripts and interview field notes multiple times and then drafting a pen portrait (Brett, 2019) for each participant. The pen portrait included background information, a narrative summary across both interviews and the researcher's reflections. The purpose of the pen portrait was only to provide the lead researcher with a comprehensive understanding of the whole story of each participant and to improve the understanding of the narrative from the participant's perspective when interpreting the narratives (Brett, 2019; Hollway & Jefferson, 2000).

The second stage involved analysing the data using thematic narrative analysis (Riessman, 2008) to explore the shared and unique experiences found across the narratives. The data were inductively coded using NVivo 12 and following Riessman's four stages of the thematic model: (1) selection of segments that are relevant to the research question; (2) developing and defining the identified thematic categories by looking closely for patterns and meanings produced by the data; (3) organising segments into the categories; and (4) drawing conclusions by describing the meaning in the content and highlighting similarities and differences across the narratives (Frost, 2021; Riessman, 2008). Throughout this stage and as part of the iterative analysis process, transcripts were repeatedly revisited to consolidate the final themes. In thematic narrative analysis, the analyst thrives to “keep the story intact for interpretive purposes” (Riessman, 2008, p.72); therefore, participants' pen portraits were regularly considered when interpreting the findings.

### Quality, rigour and reflexivity

To ensure the trustworthiness of the findings, we followed the four key criteria suggested by Lincoln and Guba (1986) to assess the rigour of the findings. At the start, the lead researcher involved the research team in designing, planning, and conducting the study through discussions during regular supervision meetings. Concerning the analysis, the lead researcher discussed pen portraits and narratives selected from each participant with another member of the research team who is a clinical psychologist and experienced in narrative research in the area of self‐harm and suicide. The lead researcher also involved the research team in the analysis processes (i.e., discussion of themes elicited from participants' stories, refining the codes, discussing developed themes, and checking the validity of the findings' interpretation).

The lead researcher is a postgraduate researcher with previous clinical experience in mental health counselling. The researcher had a specific interest in NSSI besides other mental health difficulties that are commonly linked to childhood trauma. The research team consisted of clinical psychologists, a consultant psychiatrist, and a professional expert in qualitative research. The team have clinical expertise in collaborating with individuals with NSSI and/or academic interests in self‐harm and complex trauma. One of the team members also has lived experience with NSSI. With a background in clinical mental health counselling, the analyst (lead researcher) was aware of an assumption that trauma would be a key factor driving the occurrence of NSSI as this shaped the formulation of the research question. However, this may have influenced the analyst to overlook alternative explanations or understandings of NSSI. The analyst tried to stay mindful of broader understandings by first revisiting the transcripts and the pen portraits for each participant multiple times during data analysis and results write‐up to make sure participants' whole accounts were considered when interpreting and reporting the findings. Second, the analyst involved the research team data in data interpretation and reporting.

## RESULTS

### Sample characteristics

Eight individuals (aged 20–56 years) who met the eligibility criteria participated in the study. Participants' demographic information is outlined in Table 1. All presented names are pseudonyms. Among participants, the most common method of NSSI was cutting and hitting. Childhood sexual and physical assault were the most common traumatic experiences. For C‐PTSD symptoms, the highest scores were observed in avoidance and negative self‐concept. More details about participants' scores are presented in Table 2.

**TABLE 1:** Participants demographics.

Pseudonym	Age	Gender	Ethnicity	Employment status	Age of first NSSI episode	Age of recent NSSI episode
Sarah	22	Female	White	Unemployed	10	22
Laura	26	Female	Asian	Full time	9	26
Emily	46	Female	White	Unemployed	15	46
Rebecca	47	Female	White	Unemployed	13	47
Amanda	29	Female	White	Part‐time	12	24
Andrew	56	Male	White	Unemployed	14	55
Helen	55	Female	White	Part‐time	27	54
Olivia	22	Female	White	Student	16	22

*Note*: All names are pseudonyms to preserve anonymity.

**TABLE 2:** Participants Characteristics Regarding C‐PTSD, negative life events and NSSI (*n* = 8).

C‐PTSD cluster	Score mean (*SD*)
Re‐experiencing	5.75 (2.19)
Avoidance	6.38 (1.51)
Arousal	6.13 (1.73)
Affective dysregulation	6.13 (1.46)
Negative self‐concept	6.38 (2.33)
Disturbances in relationships	5.88 (2.23)
**Non‐interpersonal negative life events**	***N* (%)**
Fire or explosion	3 (37.5%)
Transportation accident	5 (62.5%)
Serious accident	3 (37.5%)
Exposure to a toxic substance	1 (12.5%)
Assault with a weapon	4 (50%)
Combat or exposure to a warzone	1 (12.5%)
Captivity	2 (25%)
Life‐threatening illness or injury	3 (37.5%)
**Interpersonal negative life events**	***N* (%)**
Physical assault	6 (75%)
Sexual assault	6 (75%)
Other unwanted or uncomfortable sexual experiences	5 (62.5%)
Severe human suffering	3 (37.5%)
Sudden violent death	3 (37.5%)
Serious injury, harm, or death you caused to someone else	4 (50%)
**Any other very stressful event or experience**	**5 (62.5%)**
Childhood emotional abuse and neglect	2 (25%)
Drug abuse	1 (12.5%)
Parent's self‐harm	1 (12.5%)
Serious non‐life‐threatening physical injury	1 (12.5%)
**NSSI method**	***N* (%)**
Cut or carved the skin	7 (87.5%)
Burned the skin	5 (62.5%)
Inserted sharp objects into skin or nails	1 (12.5%)
Picked areas of the body to the point of drawing blood	3 (37. 5%)
Hit self on purpose	6 (75%)
Gave self a tattoo	1 (12.5%)
Scraped the skin to the point of drawing blood	5 (62.5%)

### Thematic narrative analysis results

To preserve the sequence of the life experiences, we tried to organise the themes in the same sequence as they appeared in the participants' narratives (Riessman, 2008). However, it is important to note that in many cases, these factors and experiences are intertwined with each other in a complex way. The findings are organised using the following main narrative themes: (1) Voiceless, invisible, and out of control within the dysfunctional system during childhood, (2) “Shaky foundations” leading to future traumas, (3) the link between complex trauma, mental health difficulties and NSSI, and (4) regaining autonomy and a sense of control in managing NSSI. Whilst participants' narratives were unstructured and varied considerably, they typically followed a chronological order from childhood to the present, and we have mirrored this in the ordering of the themes.

#### Voiceless, invisible, and out of control within the dysfunctional system during childhood

Complex trauma was often not a series of discrete events but was instead embedded within a wider dysfunctional system that typically originated in the family. These dysfunctional systems denied young people access to support or care or the means to make sense of and express their feelings following trauma. There was a strong sense of “being out of control and unsafe” (Rebecca) and being voiceless or unable to express one's pain, which pervaded this system. Therefore, NSSI became the only way of bringing control or expressing these feelings. However, the use of NSSI was, often punished, reinforcing this system and the sense of voicelessness and invisibility many participants experienced. This, in turn, eroded trust in others. Participants reflected on how much they constantly felt “invisible” and “voiceless” because they were not listened to nor allowed to speak up about their feelings, leading to this loss of faith or trust in others: “it's always just been fend for yourself.” (Sarah). NSSI, therefore, was the only coping strategy that was available for the person within this dysfunctional system.

Because everything around you is such a mess and you can't control anything that's going on around you at that point in time, I felt that I was in control, and doing something like that [self‐injury] you're releasing a lot of anger and stuff, and then when you've done it, you kind of feel I've got it. I'm in control here, yeah, that was my decision… (Rebecca)

…every time I spoke to my mum about how I felt about what was going on, either I got hurt or someone else got hurt. I think a lot of the, a lot of the time being told that like maybe arguments were my fault and things, so in a way it was kind of a punishment, sometimes, at the beginning to myself, and then a, a release… (Amanda)

I just never got support from anybody. I just hurt myself because I had so much inside me that I wanted to let it out and then even doing that I was still punished for my feelings and that has been a massive thing throughout my whole life. I feel like I have never been allowed to feel the way I feel. (…) knowing that you want to hurt yourself and being shouted at for it, for feeling that way, it really, really damages you in the long run, it makes you just not want to tell anyone anything, it makes you not trust anybody. (Sarah)

The experience of childhood adversity within this dysfunctional system had pronounced psychological effects. The participants Amanda, Laura, Rebecca, and Sarah further narrated how growing up in such an environment, where they constantly felt unloved, rejected, abandoned, and “let down” by close people, influenced their sense of belonging and fostered negative views of the self as being worthless and others as untrustworthy. Therefore, they learned to rely on themselves for help. Living in this dysfunctional environment from an early age appeared to be a “total” experience, affecting every aspect of participants' lives growing up, as reflected in Laura's words “It was just like bullying from every angle and like I was bullying myself by like cutting myself and punching myself” and also in Sarah's words below: “never felt home anywhere” and “Why doesn't anybody love me”.

Obviously, it [self‐injury] was quite a bit to do with the name calling off my mum, the comments about my appearance, it was just a complete worthless feeling of just, why doesn't anybody love me, why doesn't my mum love me, why aren't people at school who have a, a role of care, noticing that I'm not ok, and I've never, I've always said throughout my life, I've never felt home anywhere, like I've never had that sense of belonging (…) it's always just been fend for yourself really, nobody's gonna be there to pick you up, just do it yourself. (Sarah)

The narrative of growing up and being trapped in a dysfunctional environment was common among participants with an early onset of NSSI compared to participants whose NSSI started later. This might signify the role of a dysfunctional environment in predisposing the child to an increased risk of using NSSI at an early age.

#### “Shakey foundations” leading to future traumas

Across narratives, early exposure to complex trauma within a dysfunctional system was recounted as a factor that left participants especially vulnerable to future trauma and adversity. Laura reflected on this as having “shaky foundations”.

When I look at the end picture, so like if I looked at me as a story and on, and examining the end of my story, how I am now, in the age that I'm on now, that has totally moulded my life, the traumas and the experiences have made me addicted to drugs, they've made me drink too much, they've made me take risks that I wouldn't necessarily take, they've made me, not trust anyone, (…) it kind of just latches onto the next experience and the next experience. (Rebecca)

Not being able to make sense of earlier traumas was a factor in increasing the risk of future trauma. Amanda's narrative about how her lack of understanding of the sexual assault she had as a child led her not to report “the grooming stuff” in her teenage years, thinking she was the one to blame. The vulnerability to engaging in toxic adult relationships leading to further adversity was another common narrative. Here is Sarah narrating about how early abuse impacted her ability to form healthy adult relationships:

I feel like I've just been passed from pillar to post from being younger, and that really affected me as I am now, at being able to create relationships with people that are, that proper relationships that are good relationships that have healthy attachments, that's not something I can do. Even if something is really poisoning, really toxic, I will just latch myself onto it because I want to love something so much (…) I don't actually know, what is good and what is bad, what is healthy and what's not, because I have grown up in such a toxic environment.

Later traumas could trigger memories of childhood trauma and NSSI. Emily, for example, spoke about how she found the sexual abuse from her ex‐partner a trigger for childhood traumas and associated feelings of rage and disgust, and then NSSI.

It just, not only was it bad what he'd done, it had just then brought everything up from my childhood and so, the attacks to myself with self‐injury were not just to do with that, were to do with all that, which brought up loads of rage, which is why I would slash my whole body to pieces.

Across these narratives, recurrent trauma was linked to NSSI. The repetitive patterns of abuse from others and towards the self appeared to lock the person in a negative cycle of pain, maintaining self‐injury.

#### The link between complex trauma, mental health difficulties and NSSI

Across participants' narratives, the presence of mental health difficulties appeared to exacerbate the existing experience of “voicelessness” and “invisibility” as they were treated negatively by others: “I was treated like a sad case of a hysterical person.” (Laura) and not understanding their suffering “I am in all this pain, and no one can see it” (Amanda). Those experiences exacerbated feelings of low self‐worth and fostered social isolation, leading to a withdrawal into the self. NSSI then helps to manage these feelings and experiences as it offers a way to escape, change, or control the experience. Participants reported a wide range of mental health difficulties, often related to C‐PTSD and tied to their past traumatic experiences that were associated with their self‐injury. However, whilst participants described similar mental health difficulties (e.g., dissociation, flashbacks, avoidance, voices, low self‐worth, and relationship difficulties), the way these fed into NSSI often varied, reflecting different functions of NSSI. Helen, for example, described how self‐injury in response to the flashbacks helped her to ground herself, whereas self‐injury in response to the derogatory voices reflected a form of self‐punishment arising out of feelings of worthlessness:

The flashbacks can happen in such that, I go back to the whatever the memory is, and I use self‐injury to bring myself back to the present. ‘cos I'm so scared in my flashback and so distressed, I know that self‐injury will have quite a rapid impact on me and will rapidly bring me back, and it's very similar with the voices, the voices, if I'm very distressed by them the self‐injury will help with the distress. But also, the voices tell me to self‐injure; they talk about, the voices are derogatory and about me being the devil, evil… The voices talk about my self‐worth and that I'm worth nothing, and self‐injury is the solution to that self‐worth problem…

Olivia described similar problems of dissociation, depression, fear, low self‐worth, and “self‐inflicted loneliness”, which she linked together. However, NSSI was mostly used to deal with dissociative feelings (i.e., skin like rubber) when it first started. Olivia had a stable period where she engaged in NSSI occasionally as she managed to avoid trauma triggers. However, after moving back to her hometown (where earlier traumas occurred) and with the COVID‐19 lockdown, nightmares and flashbacks re‐occurred, leaving her feeling out of control, and her NSSI increased as she tried to control these experiences.

Then lockdown happened, and I started getting these nightmares and flashbacks (…) all these memories start crashing through, and just the stress of lockdown being back in my home town for the first extended period, then yeah, that's when the self‐harm issues started getting worse again (…) but again there's kind of like the hitting and the pinching, especially during flashbacks, the hitting was especially bad, just ‘cos I didn't feel like completely control of myself (…) my arms where kind of flying especially hitting myself in the face.

Even where NSSI was connected to similar mental health difficulties, the nature of this connection and the function of the self‐injury varied. Emily, Helen, and Amanda reported that NSSI brought them back to a conscious state after being dissociated, whilst Sarah and Amanda found NSSI helped them to dissociate from their surroundings during distress: “Everything around me'd just disappear for that amount of time” (Amanda).

#### Regaining autonomy and a sense of control in managing NSSI

Experiences of managing NSSI were common in participants' narratives, reflecting ongoing attempts to reduce or stop NSSI. Managing NSSI was a challenging process for the majority reflecting the difficulties compounded by the severity of past traumas, the presence of ongoing mental health difficulties and the reliance on NSSI over a long time. For instance, Sarah, who managed to stop NSSI only when she was pregnant, attempting to protect her baby from potential distress, found managing her NSSI very difficult, linking this challenge to the entrenched nature of her negative thoughts. Her word “re‐programme” in the following quote reflects the profound impact of early trauma on her life, reflecting once again the idea of “shaky foundations” within childhood but now affecting her as an adult.

When those thoughts and feelings start at that young an age, that young of an age that it's bound to mess you up as an adult, like I mean like your brain's so delicate, and all, all that's being put into it is negativity and all these negative feelings, it's, it's difficult to get out of that. And for me as myself, as an adult, now it's getting, it's hard to re‐programme, but I'm thinking!

Fears of negative consequences (e.g., fear of a child being taken away) at times drove efforts to reduce NSSI, but the effect was often temporary. Across all narratives, the longest‐lasting impact on NSSI came from factors that enhanced individuals' autonomy and a sense of control both internally (i.e., controlling mental state) and externally (e.g., improved living conditions). This could be seen as regaining the control that was often missing earlier in participants' narratives.

Participants spoke about several factors that helped them regain internal and external control over NSSI and other mental health difficulties. Regarding internal control, psychological therapy was one of the main factors that participants identified. Through therapy, participants were able to make sense of their experience, which helped them improve their mental state (e.g., not feeling guilty over childhood abuse). Across all stories, therapy seemed to provide participants with the language and concepts that enabled them to construct their stories and improve their understanding of their own experiences. For some, therapy provided the means of reducing mental health difficulties which in turn decreased the likelihood or severity of NSSI. For example, Olivia found the Cognitive Behavioural Therapy (CBT) techniques centred on reducing PTSD symptoms severity (alongside her relationship with her partner) helpful in reducing the severity and frequency of NSSI. Being able to control her PTSD experiences for Olivia was key.

It's the fact that I know that I have some control over my PTSD, which through the skills, it makes me feel like I'm more in control of my life, and that kind of can translate into other areas of my life (…) because I had that confidence within my mental control, my mental illness, that confidence, I feel like, has translated into other areas of my life, I feel like I'm a lot more social, I don't feel as scared meeting new people, I feel more confident like in my self‐worth as well. (Olivia)

Regaining external control, participants spoke about the positive impact of improved living conditions and quality of life on their mental well‐being. In Amanda's story, living independently, pursuing education, and adopting a healthy lifestyle improved her mood, self‐worth, and sense of agency. Those factors also allowed her to learn how to express herself without the need for NSSI. The concept of *regaining* control is important again here.

I think I had a lot more control over my own life, I think that has a lot to do with it as an adult. I had my own place to live because a lot of the time when I was self‐harming in my teenage years, I had no autonomy over my own life; a lot of the time, I had nowhere to live So, in terms of everything externally being out of control, in a sense of my security being compromised, that factor wasn't there anymore; I had a stable place to live, I was in charge of my own life, I was back in education, I was, actively in counselling and trying to be, gain some kind of recovery from, myself in a way, and I had a goal and focus, I were at uni, so, I had something to channel all my energy into, that's not to say that I didn't struggle in them times. I think, education, I got a lot of my worth from that, do you know, of feeling like, I am a good person, or that I'm loved, do you know, that was a big protective factor for me

For some, living a meaningful life where they feel valued, loved and autonomous strengthened their capability to fight against the urge to self‐injure during challenging times. For instance, Helen, who perceived her physical disability and psychosis as limiting her coping capability, found living a meaningful life “led by her” through a fulfilling job and living with a supportive, loving partner were important factors that enhanced her self‐worth and reduced her NSSI:

For me, it's about having a meaningful life rather than recovery, because I'm never gonna recover from being a voice hearer, and such that I'm on about 4 or 5 different medications ‘cos it's treatment‐resistant, but I can have a meaningful life, and having a meaningful life, doing things like this [fulfilling job] give me a reason to get up on a morning and a purpose and being busy helps with the self‐injury (…) The most positive, and this isn't about psychological therapy, the most positive influence around my self‐injury, has been the start of my new relationship with my partner, he has been a stabling influence in relation to the self‐injury (…) being happy and feeling wanted is powerful, isn't it!

Thus, even when mental difficulties persisted, a greater sense of control and autonomy in other areas was important to participants with regard to managing their self‐injury. Factors that gave participants back a “voice” to express their feelings and make sense of their experiences, made them “visible” by feeling valued, loved, and cared for, and enhanced their sense of agency by controlling their own experiences were all important. These factors reflected needs that were missing within the dysfunctional system that often pervaded their childhood.

## DISCUSSION

To our knowledge, the current qualitative study is the first to explore the complex dynamics of life events and conditions, including complex trauma, which influenced NSSI over time, as narrated by adults who screened positive for C‐PTSD. The findings showed how complex traumas occurred within a toxic caregiving milieu that leaves the individual voiceless and lacking control, created a cycle of pain triggered by trauma and reinforced by the use of NSSI. Growing up in such an environment creates a “shaky foundation” (vulnerabilities) and increases the risk of this cycle of pain being repeated across the person's life via increasing the vulnerability to further trauma, C‐PTSD‐related difficulties, and the use of NSSI as the way of coping. The difficulties of C‐PTSD and NSSI intertwined but the functions of NSSI varied between and even within individuals. Regaining autonomy and a sense of control played a vital role in reducing the severity of these difficulties. These findings are reflected in the following themes: (1) Voiceless, invisible, and out of control within the dysfunctional system during childhood, (2) “shaky foundation” leading to future trauma, (3) the link between complex trauma, mental health difficulties and NSSI, and (4) regaining autonomy and a sense of control in managing NSSI.

The study supports the established association between childhood trauma and NSSI (Christoffersen et al., 2015; Di Pierro et al., 2012), but also expands on it in several important ways. Traumatic experiences often occur within a wider dysfunctional system that compounds their impact by hindering young people's capacity to manage these experiences and denying them control and the means to express their experiences. The role of these additional family‐based risk factors (e.g., relationship quality), which often co‐occur with childhood interpersonal trauma and their link to NSSI have been supported by past research (Martin et al., 2016; Young et al., 2017). Our findings advanced this understanding by providing a nuanced exploration of how complex traumas which occurred within a toxic, dysfunctional system have set up the foundations for further trauma, moulding individuals' lives and influencing their ways of coping. Whilst NSSI provides a means to cope and respond to the harm caused by the traumas, it can inadvertently perpetuate the cycle of harm, increasing the risk of further trauma and mental health problems that are associated with C‐PTSD. Consequently, when considering the relationship between complex trauma and NSSI, these results highlight how we should consider both the wider system surrounding traumatic experiences and also how this system perpetuates further adversity over time. Within this system, NSSI is not a static endpoint but can reciprocally feedback on the adversity one faces. Future longitudinal and narrative research could further explore the reciprocal process between complex trauma, C‐PTSD and NSSI and the factors that facilitate recovery from these difficulties.

Other mental health difficulties and associated symptoms could develop as a result of trauma and the broader dysfunctional system surrounding it, which further contribute to the development of NSSI. Many previous cross‐sectional studies support the mediating role of various C‐PTSD‐related experiences (i.e., PTSD experiences, negative self‐concept, emotion dysregulation) in explaining the link between childhood interpersonal trauma and NSSI (Andersson et al., 2022; Howard et al., 2017), our work expands this understanding by illustrating how these relationships are complex and idiosyncratic with NSSI serving different functions in relation to different C‐PTSD‐related experiences. Moreover, our findings suggest that the aetiology of NSSI among people with complex trauma and C‐PTSD is not only heterogeneous, considering the severity of trauma, other contextual risk factors (e.g., lack of support, physical disability), the complex dynamics of mental health difficulties, and functions of NSSI, but also the drivers of NSSI change over time to reflect changes in life circumstances and/or mental state that carry the individual towards or away from NSSI. Recent research has shown that NSSI can serve multiple functions and that these can change over time (Taylor et al., 2023). This study illustrates how, with regard to mental health difficulties such as C‐PTSD, NSSI can be linked to particular symptoms and experiences but with idiosyncratic and varying functions. This suggests that rather than a single putative causal process existing between mental health symptomology and NSSI, multiple co‐occur processes may exist that vary between individuals and over time.

In addition to exploring the development and maintenance of NSSI, our findings also shed light on what helped people overcome these difficulties. The importance of autonomy and control in terms of recovery from NSSI was identified in the current study and is consistent with past research (Bradley et al., 2024; Kruzan & Whitlock, 2019). This study provides a useful longitudinal perspective on these changes. It was only in adulthood that participants were able to break free of the dysfunctional system they had been caught in as children and regain greater control over their lives, whether through therapy, relationships or engagement in meaningful activities. A previous qualitative study on the process of change in NSSI by Kruzan and Whitlock (2019) found that supportive relationships, learning new skills and self‐awareness were factors that facilitated change in NSSI. However, in our study, participants narrated those factors more specifically within the context of an enhanced sense of agency, self‐worth, and well‐being. The role of enhanced self‐worth as a barrier to NSSI was highlighted in the theoretical model of benefits and barriers of NSSI by Hooley and Franklin (2018), and a recent network analysis found that the lack of self‐worth was a central aspect of C‐PTSD (Knefel et al., 2019) confirming its importance as a treatment target.

Despite the important findings in the current study, limitations should be noted. Even though most of our participants were struggling with NSSI, excluding those who were at high or imminent risk of serious harm (i.e., with severe self‐harm and/or suicide ideation with the active plan) may have skewed the sample and meant the voices of those struggling to a greater extent in the present were missed. However, this was an important safety procedure considering the potential distress that may occur as a result of narrating traumatic experiences. The presented narratives were primarily from white female sample; therefore, future research should consider a more diverse population to provide a detailed account of the influence of other contextual factors (e.g., gender, ethnicity, and culture). Regarding C‐PTSD diagnosis, only one participant reported receiving a formal diagnosis, although all participants screened positive for probable C‐PTSD based on the ITQ. The lack of a formal diagnosis may influence participants' awareness of the role of C‐PTSD in relation to NSSI. However, this may also mean that participants' perceptions of how trauma shaped their NSSI were less affected by diagnostic assumptions. Since all participants were actively engaged in therapy, this may have influenced their language to draw connections between these difficulties in their story. Memory in the context of PTSD appears inconsistent, with memory deficits and difficulty voluntarily accessing specific memories being apparent alongside the phenomena of involuntary recall of vivid trauma memories (Brewin, 2007; Khan et al., 2021). This study focused on participants' autobiographical accounts and, therefore, relies on participants' memory and recall of events. Evidence suggests that narrative accounts of traumatic events have a greater abundance of sensory and perceptual details in people with PTSD and are more fragmented or less coherent (Brewin & Field, 2024; Crespo & Fernández‐Lansac, 2016). In the current study, it was possible to identify coherence and logic in participants' accounts. However, the focus here was on participants' lives more broadly and how trauma has shaped this, rather than giving accounts of traumatic events in detail, and it is possible autobiographical memory was less affected by these broader details.

The study highlights the importance of understanding the idiosyncratic nature of NSSI and trauma and that NSSI may link to trauma and C‐PTSD in different ways for different people at different times. Therefore, there is a need for a flexible and personalised approach that takes into account how NSSI fits into the individuals' unique life circumstances and experiences. A meta‐synthesis of qualitative research concerning therapy for NSSI has similarly highlighted how a nuanced and idiographic understanding of a person's self‐injury, which also recognises the person beyond the self‐injury, is important (Haw et al., 2023). Formulation‐led approaches that can tailor intervention to the individual are therefore likely important. Karatzias and Cloitre (2019) proposed a flexible multi‐modular approach to treat C‐PTSD, which appears to be promising as it allows therapists to design a treatment plan that is tailored to the individuals' specific needs. Moreover, it offers an ongoing assessment of these needs during the treatment course, allowing for a reasoned adjustment in the interventions' focus (Karatzias & Cloitre, 2019). Such an approach allows for addressing individuals' needs that change over time and helps foster individuals' autonomy through their active involvement in treatment decision‐making. Additionally, our findings highlight how the long‐lasting use of NSSI to manage internal and external experiences and its utility of serving several functions across the lifespan created a reliance on using it as a first‐line coping strategy to deal with painful experiences. Thus, adopting a non‐reductionist approach to NSSI recovery (Claréus et al., 2021) would be important to take on when formulating a treatment plan. This might start with identifying needs for NSSI and setting up a realistic expectation of NSSI recovery at the start of the treatment journey with the aim to prioritise these needs and empowering the individuals to meet them using other means of coping (Claréus et al., 2021).

Our findings highlight how helping individuals regain a sense of control or autonomy over their lives may be an important mechanism within therapy, and approaches that help foster this may be especially important. This may reflect a “non‐specific factor” that applies to many different therapies. Further research on how this sense of regained control can be fostered within therapy for those who have experienced complex trauma is needed. Moreover, attention should be paid to individuals' broader life circumstances, their relationships and roles outside of the therapy room, given the importance of these in helping people reduce their NSSI.

As our findings highlight the role of early dysfunctional caregiving milieu in the onset and maintenance of NSSI and other mental health difficulties, effective policies around identifying and preventing childhood abuse are very important. Effective prevention and early intervention programs for families with an identified risk of abuse are crucial to support children's mental health and reduce the risk of engaging in NSSI and other risky behaviours (see Bartlett et al., 2017).

## CONCLUSION

Our findings support the role of early exposure to complex trauma in increasing the vulnerability to developing mental health difficulties including NSSI. However, they also illustrate how complex trauma often occurred within a wider dysfunctional system, which compounded the difficulties participants faced, and perpetuated itself by leaving individuals vulnerable to future adversity. NSSI formed a part of this system but was reciprocally linked to trauma and adversity. Regaining a sense of control in adulthood was seen as an important process in changing one's relationship with NSSI. Therapists may therefore benefit from recognising the role and impact of the wider system surrounding particular traumatic events. Helping individuals regain a sense of control may represent an important mechanism within therapy. As individuals' mental health difficulties are changing and evolving, flexible and person‐centred treatment should be adopted to meet the complex needs of these individuals.

## AUTHOR CONTRIBUTIONS

**Reem Alharbi:** Conceptualization; investigation; writing – original draft; methodology; writing – review and editing; software; formal analysis; project administration; data curation. **Susanne Langer:** Methodology; formal analysis; writing – review and editing. **Cheryl Hunter:** Writing – review and editing; methodology; formal analysis. **Nusrat Husain:** Conceptualization; methodology; writing – review and editing; supervision. **Filippo Varese:** Conceptualization; methodology; writing – review and editing; supervision. **Peter James Taylor:** Conceptualization; writing – review and editing; methodology; supervision; formal analysis; project administration.

## CONFLICT OF INTEREST STATEMENT

There is no conflict of interest to declare.

## Data Availability

The in‐depth and detailed nature of the qualitative data supporting the findings means that even anonymised data can pose a risk of participants being identified. Therefore, it would not be appropriate to share the data.
